# Marine Lectins in Innate Immune Modulation: Mechanistic Insights, Signaling Pathways, and a Cross-Taxa Evidence Landscape

**DOI:** 10.3390/md24030102

**Published:** 2026-03-06

**Authors:** Chang-Eui Hong, Su-Yun Lyu

**Affiliations:** 1 College of Pharmacy, Sunchon National University, Suncheon 57922, Republic of Korea; gruni80@naver.com; 2 Smart Beautytech Research Institute, Sunchon National University, Suncheon 57922, Republic of Korea; 3 Research Institute of Life and Pharmaceutical Sciences, Sunchon National University, Suncheon 57922, Republic of Korea

**Keywords:** marine lectins, immunomodulation, innate immunity, macrophage activation, cytokine production, MAPK signaling, NF-κB pathway, endotoxin tolerance, antiviral activity, cancer immunotherapy

## Abstract

Marine lectins function as pattern recognition receptors in innate immunity through carbohydrate-binding mechanisms. However, mechanistic evidence detailing intracellular signaling cascades (e.g., MAPK/NF-κB/JAK-STAT activation linked to defined cytokine outputs) remains taxonomically uneven. Bivalve mollusks—particularly the Mytilectin family—represent the most extensively characterized group, whereas lectins from other marine phyla (echinoderms, cnidarians, fish, algae) have been studied primarily for structural and glycan-binding properties alongside phenotypic antimicrobial outcomes. Signaling-level resolution in native immune-cell contexts, while present in some cases, remains comparatively limited. This review synthesizes mechanistic insights dominated by bivalve-derived lectins, while integrating cross-taxa comparisons at evidence-supported levels. Specific bivalve lectins induce macrophage activation and pro-inflammatory cytokine production through reactive oxygen species-dependent activation of key signaling pathways including MAPK, NF-κB, and JAK-STAT cascades. These lectins exhibit context-dependent properties, promoting inflammatory responses in resting cells while inducing endotoxin tolerance in pre-activated macrophages through epigenetic reprogramming. Functional outcomes include broad-spectrum antiviral activity through viral envelope glycoprotein binding, anti-inflammatory effects in pain models, and cancer-associated immune responses through tumor glycan recognition and macrophage polarization. Critical gaps include uncharacterized effects on adaptive immunity, limited understanding of dendritic cell and natural killer cell interactions, and incomplete evaluation of cancer immunotherapy potential. Future research should prioritize mechanistic characterization of marine lectin-based immunotherapeutics.

## 1. Introduction

Innate immunity relies on the recognition of conserved molecular patterns to discriminate between self and non-self, enabling rapid defense against diverse pathogens [1]. Pattern recognition receptors (PRRs) detect pathogen-associated molecular patterns (PAMPs) shared by broad classes of microorganisms, forming the foundation of innate immune responses [2]. Among these PRRs, lectins—carbohydrate-binding proteins—play a central role in immunity by recognizing specific glycan structures on the surface of pathogens and host cells [1,2]. Glycans and their complementary glycan-binding proteins constitute essential components of cell–cell interactions in immune responses [3]. The human glycome comprises diverse glycan structures built from a limited set of monosaccharide building blocks, which are combined through specific biosynthetic pathways to generate functionally distinct carbohydrate patterns recognized by lectins [3]. Lectin-mediated carbohydrate recognition involves specific molecular interactions, including hydrogen bonding with sugar hydroxyl groups, CH-π interactions with aromatic amino acids, and in some cases, coordination to metal ions such as calcium [4]. These molecular interactions enable lectins to function as pattern recognition molecules, initiating and modulating various immune responses ranging from pathogen opsonization and complement activation to regulation of inflammatory processes and adaptive immune responses [1,2].

Given the fundamental role of lectins in immune recognition and regulation, marine organisms represent a particularly rich and diverse source of these carbohydrate-binding proteins. Lectins have been identified in more than 300 marine species spanning cyanobacteria, algae, invertebrates, and fish, and are classified into several structural families including C-type lectins, F-type lectins, galectins, intelectins, and rhamnose-binding lectins based on their carbohydrate recognition domains [5]. Marine invertebrate lectins function as pattern recognition molecules in innate immunity, with structural features such as signal peptides characteristic of immune-related proteins in both invertebrates and vertebrates [6]. Compared to terrestrial lectins, many marine lectins exhibit distinctive properties including low immunogenicity, relatively small molecular size, and enhanced structural stability through extensive disulfide bridge formation [7]. While specificity for complex glycans is not unique to marine lectins—as selectins, fungal lectins, and other terrestrial lectins also recognize oligosaccharide structures—many marine lectins nonetheless demonstrate high affinity for complex glycoconjugates. These characteristics position marine lectins as promising candidates for therapeutic applications, and current research has predominantly focused on their anticancer potential, with studies demonstrating selective cytotoxicity against malignant cells while exhibiting minimal toxicity to normal cells [8] as well as their broad-spectrum antimicrobial activities against bacterial and viral pathogens [9]. Although some marine lectins modulate inflammatory responses through regulation of cytokines such as interleukin (IL)-6, IL-8, and tumor necrosis factor-alpha (TNF-α) [10], comprehensive investigation of their immunomodulatory mechanisms remains limited compared with research on terrestrial lectins [5].

Understanding the immunomodulatory mechanisms of specific marine lectins has become increasingly important in the context of modern cancer immunotherapy. While immune checkpoint blockade (ICB) targeting programmed cell death protein 1 (PD-1)/programmed death-ligand 1 (PD-L1) and cytotoxic T-lymphocyte-associated protein 4 (CTLA-4) has revolutionized cancer treatment, its efficacy remains limited by the immunosuppressive tumor microenvironment (TME) and resistance mechanisms [11]. Innate immune cells, particularly tumor-associated macrophages (TAMs), represent critical determinants of therapeutic outcomes, as TAMs constitute one of the most abundant immunosuppressive cell populations in the TME and actively promote tumor progression through facilitation of angiogenesis, metastasis, and chemoresistance [12]. TAMs typically exhibit an M2-like phenotype that supports tumor growth, and reprogramming these immunosuppressive macrophages toward a pro-inflammatory M1 phenotype has emerged as a promising therapeutic strategy to restore antitumor immunity [13]. Several marine lectins possess unique dual properties combining direct cytotoxic effects on malignant cells with the demonstrated capacity to modulate inflammatory responses through regulation of cytokines and signaling pathways, positioning them as potential candidates for innate immunity-based cancer immunotherapy [14]. This review comprehensively examines the current understanding of marine lectin-mediated immunomodulation, with particular emphasis on their effects on macrophage activation, cytokine production networks, and intracellular signaling cascades including mitogen-activated protein kinase (MAPK), nuclear factor kappa B (NF-κB), and Janus kinase (JAK)-signal transducer and activator of transcription (STAT) pathways. By synthesizing scattered evidence of marine lectin immunomodulatory activities across diverse taxonomic groups and identifying key mechanistic principles, this review aims to establish marine lectins as a distinct class of innate immune modulators and to guide future research priorities toward their therapeutic applications in cancer immunotherapy.

Although lectins have been identified across a wide range of marine taxa, the depth of mechanistic immunology evidence is not uniform. Moreover, mechanistic studies employ two distinct experimental approaches: characterization of endogenous lectin functions within source organisms (e.g., pathogen recognition by hemocyte-expressed lectins in bivalves) and investigation of immunomodulatory effects when purified marine lectins are exogenously applied to mammalian cell models. This review distinguishes these contexts throughout and specifies the tissue localization of lectins and their glycan ligands where such information is available. Detailed intracellular signaling pathway characterization (e.g., MAPK/NF-κB/JAK-STAT activation linked to defined cytokine outputs and tolerance phenotypes) is currently most detailed for a subset of bivalve-derived lectins, whereas for many non-bivalve lectins the literature more commonly reports glycan-binding specificity, pathogen recognition, and phenotypic outcomes. Systematic signaling pathway characterization in native immune cells, though not entirely absent, remains comparatively sparse relative to bivalve-derived systems. Therefore, Section 3, Section 4 and Section 5 and the pathway-oriented synthesis prioritize lectins with signaling-level evidence, while cross-taxa comparisons are provided where the data support mechanistic inference and are explicitly framed as evidence-limited when signaling resolution is unavailable.

## 2. Structural Classification of Marine Lectins

**Figure 1:**
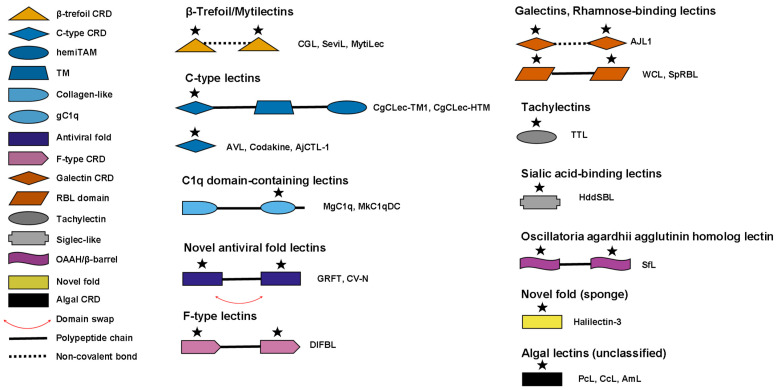
Schematic representation of domain architectures for marine lectin structural families. Left panel shows domain types and connection symbols used in this figure. Right panel displays representative domain organizations for 12 structural families with example lectins. Noncovalent dimers (CGL, SeviL, MytiLec, AJL1) are indicated by dotted lines connecting two monomers. Domain-swapped dimeric architecture (GRFT, CV-N) is indicated by bidirectional red arrows. Solid black lines represent polypeptide chains connecting domains within a single protein. Stars (★) indicate carbohydrate-binding regions. C-type lectins are shown as transmembrane receptors with hemITAM motifs (CgCLec-TM1, CgCLec-HTM) or secretory forms with single CRD (AVL, Codakine, AjCTL-1). Lectin abbreviations are defined in Table 1. Abbreviations: CRD, carbohydrate recognition domain; CV-N, cyanovirin-N; gC1q, globular C1q domain; hemITAM, half immunoreceptor tyrosine-based activation motif; OAAH, *Oscillatoria agardhii* agglutinin homolog; RBL, rhamnose-binding lectin; TM, transmembrane domain.

Marine lectins are classified into distinct structural families based on their carbohydrate recognition domains and protein architectures (Table 1, Figure 1). Among the 31 marine lectins discussed in this review, the β-trefoil/Mytilectin and C-type lectin families are most extensively characterized for immunomodulatory activities, while additional families including C1q domain-containing proteins, F-type lectins, galectins, rhamnose-binding lectins, tachylectins, and novel antiviral folds contribute to the structural and functional diversity of marine lectins. These lectins derive from diverse taxonomic sources including bivalve mollusks, echinoderms, fish, sponges, gastropods, algae, and cyanobacteria, with protein types ranging from secretory to transmembrane forms. This structural and taxonomic diversity underlies the functional versatility of marine lectins in immune recognition and modulation.

Comparative analysis reveals that marine lectins include both evolutionarily conserved families shared with terrestrial organisms and structurally unique families restricted to marine taxa. Several marine lectin families are homologous to vertebrate and plant counterparts. C-type lectins from marine invertebrates, exemplified by codakine from *Codakia orbicularis*, share amino acid sequence similarities with vertebrate selectins and natural killer cell receptors involved in cellular recognition and major histocompatibility complex interactions [6]. The β-trefoil fold characteristic of mytilectins represents the typical architecture of ricin B chain (R-type) lectins found broadly across plants, fungi, and animals [15,16]. F-type lectins have been identified in both prokaryotes and eukaryotes, including marine fish and invertebrates as well as vertebrates [17]. Galectins, characterized by β-galactoside binding specificity, are evolutionarily conserved across marine fish and mammals, participating in pathogen recognition and immune modulation in both taxa [17].

In contrast, certain marine lectin families appear to be taxonomically restricted. The mytilectin family represents a novel lectin class whose primary sequence does not match any previously described protein family, and to date has been found exclusively in species of the mollusk family Mytilidae and the phylum Brachiopoda [15,18]. Although mytilectins adopt the conserved β-trefoil fold, they exhibit distinct carbohydrate specificity compared to other R-type lectins: mytilectins bind selectively to α-galactosides, whereas fungal R-type lectins preferentially recognize β-glycosides [15]. The antiviral lectins griffithsin (GRFT), cyanovirin-N, and *Oscillatoria agardhii* agglutinin (OAA) from algae and cyanobacteria possess novel folds with no significant structural homology to known lectin families. OAA adopts a unique β-barrel topology distinct from all protein structures in the Protein Data Bank [19], while cyanovirin-N exhibits a novel fold with only distant domain-level topological similarity to known proteins [20]. These structurally unique marine lectins often display distinctive functional properties, including the picomolar antiviral potency of GRFT against human immunodeficiency virus (HIV) and other enveloped viruses [21]. These structural distinctions may underlie the unique immunomodulatory properties of marine lectins described in subsequent sections.

**Table 1:** Structural classification of marine lectins discussed in this review. Summary of 31 marine lectins organized by taxonomic group and structural family, showing their source organisms, protein types, and key structural features. Lectins are grouped to highlight the predominance of bivalve-derived lectins with pathway-level mechanistic evidence (upper section) versus comparative taxa with phenotypic-level evidence (lower section). AiL, *Argopecten irradians* lectin; AjCTL-1, *Apostichopus japonicus* C-type lectin-1; AJL1, *Anguilla japonica* lectin 1; AKL-40, *Aplysia kurodai* lectin-40; AmL, *Amansia multifida* lectin; APL, *Asterina pectinifera* lectin; AVL, *Aphrocallistes vastus* lectin; CcL, *Caulerpa cupressoides* lectin; CfL, *Chlamys farreri* lectin; CgCLec-HTM, *Crassostrea gigas* C-type lectin with hemITAM motif; CgCLec-TM1, *Crassostrea gigas* C-type lectin transmembrane protein 1; CGL, *Crenomytilus grayanus* lectin; CRD, carbohydrate recognition domain; DlFBL, *Dicentrarchus labrax* fucose-binding lectin; GalNAc, N-acetylgalactosamine; Gb3, globotriaosylceramide; GRFT, griffithsin; HddSBL, *Haliotis discus discus* sialic acid-binding lectin; hemITAM, half immunoreceptor tyrosine-based activation motif; LPS, lipopolysaccharide; MCL, *Mytilus californianus* lectin; MgC1q, *Mytilus galloprovincialis* C1q domain-containing protein; MkC1qDC, *Modiolus kurilensis* C1q domain-containing lectin; MTL, *Mytilus trossulus* lectin; MytiLec, *Mytilus galloprovincialis* lectin; OAAH, *Oscillatoria agardhii* agglutinin homolog; PAMP, pathogen-associated molecular pattern; PcL, *Pterocladiella capillacea* lectin; Q-GRFT, Q-Griffithsin; SeviL, *Mytilisepta virgata* lectin; SfL, *Solieria filiformis* lectin; SpRBL, *Strongylocentrotus purpuratus* rhamnose-binding lectin; Syk, spleen tyrosine kinase; TTL, *Tachypleus tridentatus* lectin; WCL, white-spotted charr lectin.

A. Bivalve Mollusks						
Structural Family	Lectin	Souce Organism	Taxonomic Group	Protein Type	Key Structural Features	Ref.
β-Trefoil /Mytilectin	CGL	*Crenomytilus grayanus*	Bivalve (Mussel)	Secretory	Homodimer, 6 carbohydrate-binding sites, GalNAc/Gal-specific	[22]
	SeviL	*Mytilisepta* *virgata*	Bivalve (Mussel)	Secretory	β-trefoil fold, galactoside-binding	[23]
	MytiLec	*Mytilus* *galloprovincialis*	Bivalve (Mussel)	Secretory	R-type lectin, Gb3-binding, β-trefoil fold	[24]
	MCL	*Mytilus* *californianus*	Bivalve (Mussel)	Secretory	Glycosylated, α/β fold with β predominance	[25]
	MTL	*Mytilus trossulus*	Bivalve (Mussel)	Secretory	Dimeric, 6 ligand-binding sites	[26]
C-type Lectin	CgCLec-TM1	*Crassostrea gigas*	Bivalve (Oyster)	Transmembrane	CRD + hemITAM cytoplasmic tail, Syk-associated	[27]
	CgCLec-HTM	*Crassostrea gigas*	Bivalve (Oyster)	Transmembrane	CRD + hemITAM, LPS/bacteria binding	[28]
	Codakine	*Codakia orbicularis*	Bivalve (Clam)	Secretory	Selectin-like, mannose-specific	[6]
	AiL	*Argopecten* *irradians*	Bivalve (Scallop)	Secretory	Long CRD, opsonization function	[29]
	CfL	*Chlamys farreri*	Bivalve (Scallop)	Secretory	Pathogen recognition, phagocytosis enhancement	[30]
C1q domain-containing	MgC1q	*Mytilus* *galloprovincialis*	Bivalve (Mussel)	Secretory	High sequence variability, PAMP recognition	[31]
	MkC1qDC	*Modiolus* *kurilensis*	Bivalve (Mussel)	Secretory	Antibacterial, hemal system localization	[32]
**B. Other Marine Taxa**						
**Structural** **Family**	**Lectin**	**Souce Organism**	**Taxonomic Group**	**Protein Type**	**Key Structural Features**	**Ref.**
C-type Lectin	AVL	*Aphrocallistes vastus*	Sponge	Secretory	Ca^2+^-dependent, D-galactose-specific	[10,33]
	AjCTL-1	*Apostichopus* *japonicus*	Echinoderm (Sea cucumber)	Secretory	Ca^2+^-dependent bacterial agglutination	[34]
	APL	*Asterina* *pectinifera*	Echinoderm (Starfish)	Secretory	AMPK-dependent signaling	[35]
F-type lectin	DlFBL	*Dicentrarchus labrax*	Fish (Sea bass)	Secretory	Two tandem CRDs, F-type lectin motif, fucose-binding	[36]
Galectin	AJL1	*Anguilla japonica*	Fish (Eel)	Secretory	β-galactoside-binding homodimer, Ca^2+^-independent galectin	[37]
Rhamnose-binding lectin (RBL)	WCL	*Salvelinus* *leucomaenis*	Fish (Charr)	Secretory	L-rhamnose-specific	[35]
	SpRBL	*Strongylocentrotus purpuratus*	Echinoderm (Sea urchin)	Secretory	L-rhamnose-specific, genome-derived RBL fold	[38]
Tachylectin	TTL	*Tachypleus* *tridentatus*	Chelicerate (Horseshoe crab)	Secretory	LPS-binding, innate immunity	[35]
Sialic acid-binding lectin	HddSBL	*Haliotis discus* *discus*	Gastropod (Abalone)	Secretory	Siglec-like, sialic acid-specific	[39]
Novel antiviral fold	GRFT	*Griffithsia* sp.	Red Alga	Secretory	Domain-swapped dimer, 6 mannose-binding sites	[21,40,41,42]
	cyanovirin-N	*Nostoc* *ellipsosporum*	Cyanobacterium	Secretory	Domain B glycan binding, antiviral	[43,44,45]
	Scytovirin	*Scytonema varium*	Cyanobacterium	Secretory	Mannose-rich oligosaccharide binding	[46]
	Q-GRFT	Recombinant *(Nicotiana* *benthamiana)*	Recombinant	Secretory	GRFT variant, antifungal activity	[47]
OAAH family	SfL	*Solieria filiformis*	Red Alga	Secretory	Two β-barrel domains, mannose-specific	[48]
DUF3011 family	AKL-40	*Aplysia kurodai*	Gastropod (Sea hare)	Secretory	Three repeated domains, D-galacturonic acid binding	[49]
Novel fold (sponge)	Halilectin-3	*Haliclona caerulea*	Sponge	Secretory	No similarity to known families, GalNAc-binding	[25]
Algal lectin (unclassified)	PcL	*Pterocladiella* *capillacea*	Red Alga	Secretory	5.8 kDa monomer, mucin-inhibitable	[50]
	CcL	*Caulerpa* *cupressoides*	Green Alga	Secretory	44.7 kDa dimer, lactose /galactose-specific	[51]
	AmL	*Amansia multifida*	Red Alga	Secretory	Mannose-inhibitable, anti-nociceptive	[52]

## 3. Innate Immune Cell Activation

Macrophages serve as critical effector cells in innate immunity, and marine lectins induce potent macrophage activation. While some lectins act through glycan recognition, others may utilize CRD-independent mechanisms. The mechanistic evidence presented in this section derives predominantly from bivalve-derived lectins, particularly CGL, SeviL, and MytiLec from the Mytilectin family, which represent the most thoroughly characterized marine lectins for macrophage signaling pathway activation. While lectins from other marine taxa (sponges, algae, fish) have demonstrated immunomodulatory activities, detailed characterization of their intracellular signaling mechanisms in immune cells remains limited in the current literature. A galactose/N-acetylgalactosamine (GalNAc/Gal)-specific lectin from the sea mussel *Crenomytilus grayanus* (CGL), expressed in hemolymph, possesses a dimeric quaternary structure that provides six carbohydrate-binding sites enabling multivalent glycan recognition [53]. When exogenously applied, CGL induced secretion of TNF-α and IL-6 across multiple macrophage types including mouse RAW264.7 macrophages, mouse bone marrow-derived macrophages (BMDMs), human THP-1 macrophages, human peripheral blood mononuclear cells (PBMCs), and human blood monocyte-derived macrophages (MDMs), demonstrating broad cross-species activity [22]. Notably, sugar competition assays showed that CGL-mediated cytokine production was independent of its carbohydrate-binding activity, suggesting non-CRDs contribute to immunomodulation. This cytokine production was regulated through reactive oxygen species (ROS)-dependent activation of MAPK cascades (extracellular signal-regulated kinase [ERK]1/2, c-Jun N-terminal kinase [JNK]1/2, and p38), protein kinase C (PKC)-α/δ, and nuclear factor-kappa B (NF-κB) pathways [15]. Similarly, when exogenously applied to RAW264.7 macrophages, SeviL, a galactoside-binding lectin isolated from the mussel *Mytilisepta virgata*, promoted polarization of RAW264.7 cells toward a pro-inflammatory M1 functional phenotype through both MAPK and JAK-STAT signaling pathways [23]. Marine sponge-derived lectins have also demonstrated immunomodulatory capacity, with Aphrocallistes vastus lectin (AVL), when applied exogenously, increasing production of IL-6, IL-8, and TNF-α in hepatocellular carcinoma (HCC) cells [10]. These findings collectively establish the aforementioned marine lectins as potent activators of macrophage inflammatory responses through engagement of multiple intracellular signaling cascades.

**Figure 2:**
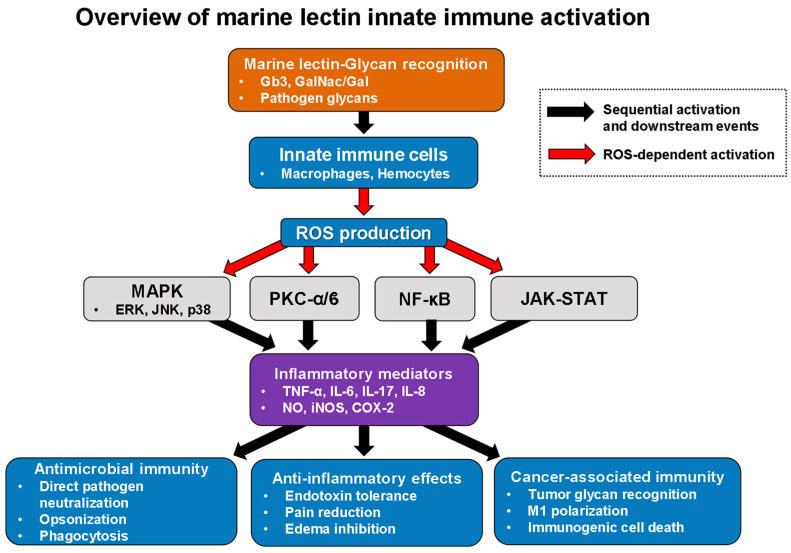
Marine lectin-mediated innate immune activation and functional outcomes. Marine lectin–glycan binding (Gb3, GalNAc/Gal, pathogen glycans) activates innate immune cells through extracellular recognition of cell surface glycans. Mechanistic evidence derives predominantly from exogenous application of bivalve-derived lectins (CGL, SeviL, MytiLec) to mammalian macrophage models (RAW264.7, THP-1), with endogenous functions characterized for oyster C-type lectins (CgCLec-TM1, CgCLec-HTM) in hemocytes. This triggers ROS-dependent activation of MAPK (ERK, JNK, p38), PKC-α/δ, NF-κB, and JAK-STAT pathways, leading to inflammatory mediator production. Functional outcomes include antimicrobial immunity through direct pathogen neutralization and phagocytosis, anti-inflammatory effects through endotoxin tolerance and pain reduction, and cancer-associated immunity through tumor glycan recognition and macrophage polarization. Mechanistic evidence for signaling pathway activation derives predominantly from bivalve-derived lectins (CGL, SeviL, MytiLec, CgCLec-TM1, CgCLec-HTM) and the sponge lectin AVL, as detailed in Table 2. Red arrows indicate ROS-dependent activation; black arrows indicate sequential activation and downstream events. COX-2, cyclooxygenase-2; DAMPs, damage-associated molecular patterns; ERK, extracellular signal-regulated kinase; Gb3, globotriaosylceramide; GalNAc/Gal, galactose/N-acetylgalactosamine; IL, interleukin; iNOS, inducible nitric oxide synthase; JAK-STAT, Janus kinase-signal transducer and activator of transcription; JNK, c-Jun N-terminal kinase; MAPK, mitogen-activated protein kinase; NF-κB, nuclear factor-kappa B; NO, nitric oxide; PAMPs, pathogen-associated molecular patterns; PKC, protein kinase C; ROS, reactive oxygen species; TNF-α, tumor necrosis factor-alpha.

Beyond cytokine induction, marine lectin-activated macrophages exhibit enhanced functional capabilities that contribute directly to antimicrobial defense. Certain marine lectins can act as opsonins and agglutination agents, facilitating pathogen recognition and clearance by innate immune cells. CGL treatment slightly but consistently increased the bactericidal activity of macrophages and induced cytokine production in mouse models, demonstrating in vivo immunomodulatory effects [22]. Interestingly, CGL displayed context-dependent immunoregulatory properties in macrophages pre-activated with bacterial lipopolysaccharide (LPS). In this context, CGL induced endotoxin tolerance characterized by downregulation of nitric oxide (NO), inducible nitric oxide synthase (iNOS), IL-6, and cyclooxygenase (COX)-2 expression through downregulation of IL-1 receptor-associated kinase (IRAK) 2, reduced JNK1/2 phosphorylation, and suppression of NF-κB activation [22]. This dual functionality—promoting pro-inflammatory responses in resting macrophages while inducing tolerance in LPS-activated cells—suggests sophisticated immunoregulatory mechanisms that prevent excessive inflammation while maintaining antimicrobial defense capacity. Marine lectin-mediated innate immune activation follows a coordinated cascade from initial ligand recognition through intracellular signaling to diverse functional outcomes (Figure 2). This integrated pathway demonstrates how these marine lectins function as pattern recognition molecules that bridge molecular recognition events to complex immunological responses spanning antimicrobial defense, inflammation resolution, and cancer immunity.

## 4. Inflammatory Mediator Production

The inflammatory mediator responses detailed in this section have been most extensively characterized in bivalve systems, where lectins such as CGL, CgCLec-TM1, and CgCLec-HTM provide the primary mechanistic evidence for cytokine induction pathways. Marine algal lectins (CcL, PcL, SfL) contribute anti-inflammatory data notably from in vivo rodent inflammatory models [48,50,51], with cellular signaling characterization in these systems being comparatively limited. This taxonomic distribution of evidence reflects current research priorities rather than inherent differences in lectin functionality across marine phyla.

### 4.1. Pro-Inflammatory Cytokines

Pro-inflammatory cytokines are essential signaling molecules that initiate and coordinate immune responses against microbial invasion. In mollusks, which rely exclusively on innate immunity for defense, these cytokines play particularly critical roles in pathogen recognition and elimination [54]. Marine lectins have been demonstrated to induce the production of several key pro-inflammatory cytokines through distinct molecular mechanisms. As described in Section 3, the GalNAc/Gal-specific lectin CGL from *Crenomytilus grayanus* potently induces TNF-α and IL-6 across multiple macrophage types through ROS-MAPK-NF-κB pathways [22]. Similarly, oncolytic vaccinia virus carrying AVL (oncoVV-AVL) increased IL-6, IL-8, and TNF-α levels in HCC cells through activator protein-1 (AP-1) pathway activation [10]. In bivalve mollusks, TNF-like molecules coordinate with the immune deficiency, Toll, and JNK pathways to control phenoloxidase activity and antimicrobial peptide expression, with the NF-κB signaling pathway being crucial for mediating cellular TNF responses and promoting cell survival through anti-apoptotic gene expression [55].

Among mollusk cytokines, IL-17 represents the most evolutionarily conserved and functionally important pro-inflammatory mediator. IL-17 homologs have been identified across diverse invertebrate phyla, including echinoderms, tunicates, and mollusks, indicating ancient evolutionary origins predating adaptive immunity [56,57]. Bivalve mollusks exhibit extensive expansion of IL-17 genes compared to other invertebrates. The mussel *Mytilus galloprovincialis* possesses 379 unique IL-17 sequences classified into 23 distinct isoforms and 96 IL-17 receptors grouped into 6 isoforms [58]. This expansion suggests that IL-17-mediated immunity plays a particularly critical role in filter-feeding bivalves constantly exposed to diverse microbial challenges.

The first molluscan IL-17 was cloned from *Crassostrea gigas* hemocytes in 2008, revealing structural similarity to vertebrate IL-17s, including conserved cysteine residues essential for the cystine knot fold. Injection of bacteria into Pacific oysters produced large and rapid increases in IL-17 transcript abundance in hemocytes, with levels highly elevated by 6 h, indicating that IL-17 functions as a very early response gene stimulating downstream immune effectors [56]. Subsequent studies confirmed that IL-17 expression is significantly upregulated in oyster and mussel hemocytes following exposure to PAMPs, including LPS, peptidoglycan (PGN), and β-glucan [59,60].

A direct mechanistic link between lectin recognition and IL-17 production has been established in oysters through studies of C-type lectin receptors. The oyster C-type lectin transmembrane protein 1 (CgCLec-TM1) contains a carbohydrate recognition domain and a hemITAM (half immunoreceptor tyrosine-based activation motif) in its cytoplasmic tail. In oyster hemocytes, CgCLec-TM1 binds LPS and bacteria through its carbohydrate recognition domain and associates with spleen tyrosine kinase (Syk) to activate ERK phosphorylation, which then interacts with glycogen synthase kinase-3β (GSK3β) to induce expression of *Crassostrea gigas* IL-17 isoforms CgIL-17-1 and CgIL-17-5 following *Vibrio splendidus* challenge [27]. Another oyster C-type lectin with hemITAM motif (CgCLec-HTM), also functioning in hemocytes, similarly induced IL-17 and TNF production through a Syk/ERK/NF-κB family transcription factor Rel signaling pathway following LPS stimulation [28]. In mammals, C-type lectin receptors regulate IL-17 production through multiple immune cell types. Dectin-2 mediates IL-17-related immune responses through activation of dendritic cells (DCs) for Th17 differentiation and direct regulation of neutrophil IL-17 production in response to fungal pathogens [61], indicating functional conservation of C-type lectin-mediated cytokine induction across phyla.

IL-17 production in mollusks requires precise regulation to balance protective immunity and immunopathology. In the thick shell mussel *Mytilus coruscus*, overexpression of *Mytilus coruscus* IL-17 isoform 1 (McIL-17-1) through recombinant protein injection resulted in significantly higher mortality rates following *Vibrio alginolyticus* infection, whereas RNA interference-mediated knockdown of McIL-17-1 reduced mortality. Flow cytometric analysis demonstrated that McIL-17-1 inhibition significantly reduced LPS-induced apoptosis in hemocytes [59]. These results indicate that IL-17 mediates inflammatory responses to bacterial infection, with excessive IL-17 production potentially causing immunopathology through enhanced inflammatory responses and increased cell death. The functional diversity of mollusk IL-17 isoforms likely reflects specialization for distinct immune challenges, as different IL-17 isoforms in *Mytilus galloprovincialis* exhibited tissue-specific expression patterns and differential responses to waterborne *Vibrio splendidus* infection in gill tissues [58].

IL-17 signaling in mollusks shares mechanistic conservation with vertebrate systems. Mollusk IL-17 activates NF-κB pathways [62], and IL-17 represents an evolutionarily conserved cytokine with conserved roles in mucosal immunity, inducing antimicrobial peptide expression and recruiting phagocytic cells [57,58]. The integration of lectin-mediated pathogen recognition with IL-17-dependent inflammation represents an important component of mollusk innate immunity. Marine lectins such as CgCLec-TM1 and CgCLec-HTM functioning as PRRs initiate signaling cascades resulting in IL-17 production, which amplifies immune responses through cytokine networks and antimicrobial effector induction.

**Figure 3:**
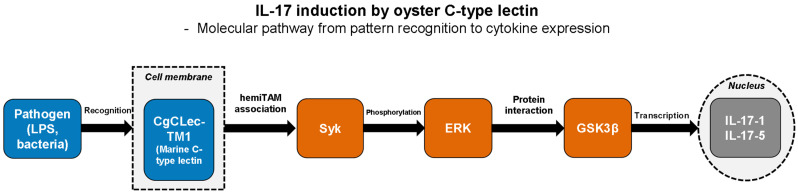
IL-17 induction pathway by oyster C-type lectin. Pathogen recognition by the transmembrane marine C-type lectin CgCLec-TM1. CgCLec-TM1 recognizes bacterial pathogens such as *Vibrio splendidus* and their LPS at the cell membrane. Upon ligand binding, CgCLec-TM1 associates with Syk through its hemITAM, initiating a phosphorylation cascade. Activated Syk phosphorylates ERK, which then interacts with GSK3β. GSK3β translocates to the nucleus and induces transcriptional activation of IL-17 genes (CgIL-17-1 and CgIL-17-5). This pathway represents an evolutionarily conserved mechanism linking pattern recognition to pro-inflammatory cytokine production in mollusk innate immunity. Arrows indicate sequential steps in signal transduction. Abbreviations: CgCLec-TM1, *Crassostrea gigas* C-type lectin transmembrane protein 1; ERK, extracellular signal-regulated kinase; GSK3β, glycogen synthase kinase-3β; hemITAM, half immunoreceptor tyrosine-based activation motif; IL-17, interleukin-17; LPS, lipopolysaccharide; Syk, spleen tyrosine kinase.

The molecular mechanism linking C-type lectin recognition to IL-17 production is illustrated in oyster hemocytes (Figure 3), where sequential activation from pattern recognition through kinase cascades to nuclear transcription demonstrates the conserved architecture of lectin-mediated cytokine induction in invertebrate immunity.

### 4.2. Anti-Inflammatory Effects

While marine lectins demonstrate potent pro-inflammatory capabilities, several species exhibit context-dependent immunoregulatory properties that encompass anti-inflammatory activities. As described in Section 3, CGL from the sea mussel *Crenomytilus grayanus* exemplifies this sophisticated dual functionality, inducing pro-inflammatory cytokines in resting macrophages while triggering endotoxin tolerance in LPS-activated macrophages through downregulation of the Toll-like receptor (TLR)-mediated NF-κB pathway [22]. This ability to promote pro-inflammatory responses in naive immune cells while simultaneously inducing tolerance in pre-activated cells suggests sophisticated immunoregulatory mechanisms that may prevent excessive inflammation while maintaining antimicrobial defense capacity.

At the molecular level, marine algal lectins suppress inflammation through inhibition of key pro-inflammatory mediators and signaling pathways. Systemically administered lectin from the green seaweed *Caulerpa cupressoides* (CcL) exhibited anti-inflammatory activities involving inhibition of IL-1β, TNF-α, IL-6, and COX-2 expression, as well as modulation of histamine H1 receptors in carrageenan-induced rat paw edema models [51]. While other marine-derived compounds such as sulfated polysaccharides, terpenoids, and fatty acids also exhibit anti-inflammatory activities, these effects occur through non-carbohydrate-binding mechanisms [63,64,65]. In contrast, the anti-inflammatory effects of these marine lectins appear to involve carbohydrate-mediated mechanisms, as evidenced by the reversal of some, but not all, effects upon mucin pre-treatment [51].

The context-dependent nature of marine lectin immunomodulation extends beyond cellular activation states to encompass administration routes. CcL demonstrates anti-inflammatory properties when administered systemically yet elicits pro-inflammatory responses when administered locally, suggesting differential interactions that may involve the lectin domain and carbohydrate residues on inflammatory cell membranes [51]. The functional anti-inflammatory effects manifest as significant antinociceptive activities in classical pain and inflammation models. Lectins from the red marine algae *Pterocladiella capillacea* and *Solieria filiformis*, when administered to rodent inflammation models, demonstrated significant anti-inflammatory and antinociceptive activities [48,50], whereas the lectin from *Amansia multifida* inhibited carrageenan-induced paw edema by 82% [52]. These findings indicate that these marine lectins exert anti-inflammatory effects through carbohydrate-dependent mechanisms, with functional outcomes extending from cellular immunomodulation to systemic pain relief and inflammation resolution.

### 4.3. Cell Adhesion and Leukocyte Trafficking

Leukocyte recruitment to inflammatory sites constitutes a prerequisite for the immune responses described above. Leukocyte extravasation from the vascular lumen into inflamed tissues involves sequential steps including rolling along the endothelium, firm adhesion, and transmigration, orchestrated by selectins and integrins [66]. Structural analyses reveal that the marine C-type lectin codakine from the clam *Codakia orbicularis* shares amino acid sequence similarities with selectins and natural killer (NK) cell receptors, which participate in cellular recognition and immune responses through carbohydrate binding [6].

The marine sponge lectin halilectin-3 from *Haliclona caerulea*, when applied exogenously to MCF-7 breast cancer cells, modulated cell adhesion through direct integrin interactions, reducing the expression of integrin α6β1 and interacting with integrin α5β1 (the fibronectin receptor), thereby impairing adhesion and promoting anoikis [67]. While these structural and functional findings suggest that marine lectins may influence leukocyte trafficking through interactions with adhesion molecules, direct experimental evidence demonstrating marine lectin-mediated modulation of leukocyte migration in vivo remains limited, representing an important area for future investigation.

### 4.4. Opsonization and Phagocytosis

Beyond regulating leukocyte trafficking, certain marine lectins function as opsonins that bridge pathogens to phagocytic cells, thereby enhancing microbial recognition and clearance. These marine lectins demonstrate broad-spectrum opsonization capabilities across diverse invertebrate taxa. In bivalve mollusks, lectins present in hemolymph and pallial fluid function endogenously as opsonins that facilitate phagocytosis by hemocytes, with oyster *Crassostrea virginica* hemolymph lectins promoting bacterial adherence to external mucosal surfaces and enhancing subsequent phagocytic uptake [68,69]. The C-type lectin codakine from the clam *Codakia orbicularis* exhibits structural homologies with selectins and NK cell lectins involved in cellular recognition, suggesting potential roles in opsonization and phagocytosis during innate immune responses [6]. The C1q domain-containing protein MgC1q from the mussel *Mytilus galloprovincialis*, functioning endogenously, functions as a pattern recognition molecule with high sequence variability suggestive of somatic diversification, enabling recognition of diverse PAMPs during innate immune responses [31]. Similarly, the C1q domain-containing lectin MkC1qDC from the mussel *Modiolus kurilensis*, functioning endogenously within the organism, demonstrated antibacterial properties against both Gram-negative and Gram-positive bacteria, localizing to the hemal system and interstitial compartments where it participates in pathogen opsonization [32]. The C-type lectin AjCTL-1 from the sea cucumber *Apostichopus japonicus*, functioning endogenously, exhibited calcium-dependent bacterial agglutination and significantly enhanced the phagocytic capacity of coelomocytes against *Escherichia coli* in vitro [34]. In scallops, lectins from *Argopecten irradians* and *Chlamys farreri*, functioning endogenously, promoted hemocyte-mediated phagocytosis through opsonization of bacterial pathogens [29,30]. Collectively, these observations establish that these marine lectins serve dual roles as PRRs that identify non-self carbohydrate structures on pathogen surfaces and as opsonins that facilitate phagocytic clearance, thereby constituting critical components of invertebrate humoral immunity.

## 5. Intracellular Signaling Pathways

The signaling pathway analyses presented in Section 5.1, Section 5.2, Section 5.3 and Section 5.4 are derived predominantly from bivalve lectins (CGL, MytiLec, SeviL, CgCLec-TM1, CgCLec-HTM) and the sponge lectin AVL. This concentration of mechanistic data reflects the current literature landscape: while lectins from fish, echinoderms, algae, and cnidarians have been identified and functionally characterized for direct antimicrobial or cytotoxic activities, systematic investigation of their intracellular signaling cascades in immune cells remains an important gap for future research.

### 5.1. MAPK Cascades

The inflammatory mediator production described in the preceding section is regulated by intracellular signaling pathways, with MAPK cascades (ERK, JNK, p38) representing critical modules through which several marine lectins activate cytokine production [22,70,71].

Several marine lectins activate all three major MAPK subfamilies (ERK1/2, JNK1/2, and p38) in immune cells. For example, CGL induces macrophage activation through ROS-dependent MAPK phosphorylation leading to cytokine production (Section 3) [22]. The β-trefoil lectin MytiLec from *Mytilus galloprovincialis*, which presents three carbohydrate-binding sites that facilitate multivalent recognition of Gb3 glycosphingolipids [15], when applied exogenously, similarly activates both classical MAPK (MEK)-ERK and stress-activated p38/JNK MAPK pathways in Burkitt’s lymphoma (BL) cells, with MytiLec-induced phosphorylation of the MEK-ERK pathway upregulating expression of the cyclin-dependent kinase inhibitor p21, ultimately leading to cell cycle arrest and TNF-α production [24]. SeviL, when applied exogenously, demonstrates dose- and time-dependent activation of the upstream kinase MKK3/6 and its downstream target p38 MAPK in cervical cancer cells, accompanied by activation of effector caspases-3 and -9 [72].

Certain marine lectins incorporated into oncolytic viral vectors demonstrate that MAPK signaling is functionally required for therapeutic efficacy. AVL expressed in oncolytic vaccinia virus, when delivered exogenously, stimulates ERK phosphorylation in cancer cells, with viral replication enhancement being completely dependent on ERK activity as demonstrated by ablation of the replication-promoting effect following treatment with the MEK inhibitor U0126 [73]. The phosphorylated ERK subsequently binds with promoters of AP-1 components c-Jun and c-Fos, inducing their expression and ultimately increasing levels of inflammatory cytokines including IL-6, IL-8, and TNF-α in HCC cells [10].

In molluscan immunity, MAPK signaling serves as a critical intermediate between lectin-mediated pathogen recognition and downstream effector responses, providing mechanistic insight into the immune functions observed in Section 4. The oyster C-type lectin CgCLec-TM1 binds LPS and bacteria through its carbohydrate recognition domain, then associates with Syk via its hemITAM motif to activate ERK phosphorylation [27]. The activated ERK subsequently interacts with GSK3β to phosphorylate it at serine 9, which induces expression of IL-17 isoforms CgIL-17-1 and CgIL-17-5 following *Vibrio splendidus* challenge. These observations collectively establish MAPK cascades as evolutionarily conserved signaling intermediates in marine lectin-mediated inflammatory responses. While several marine lectins, including MytiLec [24] and SeviL [72], exert their effects through glycan-dependent mechanisms as demonstrated by glycan-specific binding and antibody inhibition studies, CGL-induced cytokine production in macrophages has been demonstrated to occur independently of its sugar-binding property, as monosaccharide pre-incubation did not affect CGL-mediated TNF-α production [22]. These findings indicate that marine lectins activate MAPK cascades through both carbohydrate-dependent and carbohydrate-independent mechanisms.

### 5.2. NF-κB Pathway

NF-κB represents a parallel signaling pathway that coordinates with MAPK cascades to regulate inflammatory cytokine production following lectin-mediated pathogen recognition [74]. C-type lectins with transmembrane regions recognize invading extracellular pathogens via their carbohydrate recognition domains and trigger downstream cascades involving Syk, MAPK, NF-κB, and AP-1 to initiate the production of proinflammatory cytokines [27,75].

The mussel lectin CGL exemplifies direct NF-κB pathway activation in mammalian immune cells, inducing TNF-α and IL-6 secretion in mouse RAW264.7 macrophages, bone marrow-derived macrophages, human THP-1 macrophages, human peripheral blood mononuclear cells, and human blood monocyte-derived macrophages through NF-κB activation [22]. The molecular architecture connecting pattern recognition to NF-κB activation involves evolutionarily conserved signaling modules across phylogenetically distant taxa [76,77]. In the Pacific oyster *Crassostrea gigas*, the C-type lectin receptor CgCLec-HTM, functioning endogenously in hemocytes, binds LPS and various bacteria, subsequently associating with the Src homology 2 domain of Syk through a nonclassical immunoreceptor tyrosine-based activation motif in its cytoplasmic tail to promote ERK phosphorylation. Activated ERK then interacts directly with Rel to induce its nuclear translocation and subsequent transcription of IL-17 and TNF genes [28].

The evolutionary conservation of NF-κB regulatory machinery extends to upstream signaling components across invertebrate phyla. An inhibitor of κB (IκB) kinase (IKK)-like protein from Pacific oyster possesses the characteristic organization of mammalian IKK proteins, including an amino-terminal kinase domain, leucine zipper region, and carboxy-terminal helix-loop-helix motif, and when transfected into human cell lines activates NF-κB-controlled reporter gene expression [78]. In the freshwater mussel *Anodonta woodiana*, MyD88 associates with TLRs to activate NF-κB and AP-1 signaling pathways in human HEK293T cells, demonstrating functional interchangeability of invertebrate and vertebrate signaling components [79]. The Rel family of transcription factors shows parallel diversification, with insects expressing four Rel proteins in addition to Dorsal, all involved in immune responses [78].

Certain marine lectins exhibit context-dependent immunomodulatory functions through differential regulation of NF-κB pathway components depending on cellular activation state. Pre-incubation of macrophages with CGL induces endotoxin tolerance, a phenomenon characterized by reduced inflammatory responses upon LPS challenge, with this tolerance mediated by degradation of IRAK2, a downstream TLR-4 signaling molecule [80]. Induction of endotoxin tolerance increases bacterial clearance and improves survival in mice with sepsis, positioning such marine lectins as potential therapeutic agents capable of preventing excessive inflammation while maintaining antimicrobial defense capacity [22,81,82].

### 5.3. Immune Tolerance Mechanisms

Marine lectin-activated inflammatory pathways are subject to negative feedback regulation through endotoxin tolerance, a protective state wherein cells exhibit diminished inflammatory responses to subsequent stimulation despite prior activation [83]. This tolerance phenomenon prevents excessive inflammation and tissue damage while maintaining antimicrobial defense capacity [84].

Among marine lectins, CGL from *Crenomytilus grayanus* represents the only example with experimentally characterized tolerance-inducing activity [22]. Pre-treatment of RAW264.7 macrophages with CGL (10 μg/mL) for 24 h followed by LPS challenge resulted in a tolerance-like phenotype characterized by attenuation of specific pro-inflammatory mediators: IL-6 secretion and NO production were markedly reduced, while iNOS and COX-2 expression were downregulated at the protein level. Mechanistically, CGL-induced tolerance was associated with reduced IRAK2 expression, diminished JNK1/2 phosphorylation, and suppressed NF-κB activation in LPS-stimulated cells. Notably, this tolerance phenotype exhibited pathway selectivity: TNF-α production remained unaffected by CGL pre-treatment, and ERK1/2 and p38 phosphorylation were not reduced, indicating selective hyporesponsiveness rather than global inflammatory suppression [22].

The pathway-specific nature of CGL-induced tolerance parallels mechanisms described in mammalian endotoxin tolerance, where IRAK-M and related negative regulators selectively suppress pro-inflammatory gene transcription while preserving antimicrobial effector functions [85,86]. This overlap suggests that marine lectins may modulate tolerance-related signaling nodes shared across taxa. Clinical relevance is supported by observations that dysregulated IRAK family expression is associated with adverse outcomes in septic patients [87]. However, CGL currently represents the sole marine lectin with experimentally demonstrated tolerance-inducing activity. Whether other marine lectins—particularly those that activate similar MAPK and NF-κB cascades (Section 5.1 and Section 5.2)—possess comparable immunomodulatory properties remains uninvestigated, representing a priority for future marine lectin immunology research.

### 5.4. Other Pathways

Some marine lectins activate signaling pathways beyond the MAPK and NF-κB cascades described above, including the JAK–STAT pathway, which is engaged by SeviL during promotion of RAW264.7 macrophage polarization toward a pro-inflammatory M1 functional phenotype [23]. SeviL activation of JAK-STAT signaling results in enhanced expression of IL-6 and TNF-α encoding mRNAs and secretion of pro-inflammatory cytokines and chemokines [23].

The sponge lectin AVL modulates the phosphatidylinositol 3-kinase (PI3K)/Akt pathway alongside MAPK/ERK and Hippo/mammalian STE20-like protein kinase (MST) pathways through cross-talk mediated by Raf-1 kinase [88]. AVL additionally promotes inflammatory cytokine production including IL-6, IL-8, and TNF-α through activation of the activator protein-1 (AP-1) signaling pathway [10]. The coordinate regulation of PI3K/Akt, Hippo, and lipid metabolism pathways by AVL occurs through AMP-activated protein kinase (AMPK), which integrates diverse cellular signaling networks [35]. This AMPK-dependent multi-pathway integration extends to other marine lectins including *Asterina pectinifera* lectin (APL), white-spotted charr lectin (WCL), and *Tachypleus tridentatus* lectin (TTL), which similarly modulate signaling networks in cell-type-dependent manners [35].

Fish-derived lectins including *Dicentrarchus labrax* fucose-binding lectin (DlFBL) and *Anguilla japonica* lectin 1 (AJL1), when applied exogenously to cancer cells, induce apoptosis through downregulation of the E2F transcription factor 1 (E2F-1), subsequently activating Bcl-2 family apoptotic proteins and suppressing anti-apoptotic proteins including B-cell lymphoma 2 (Bcl-2) and X-linked inhibitor of apoptosis protein (XIAP) [38]. The engagement of diverse pathways including JAK-STAT, PI3K/Akt, AP-1, AMP kinase (AMPK), and apoptotic cascades by these structurally distinct marine lectins demonstrates that carbohydrate recognition serves as a versatile trigger for activating multiple intracellular signaling networks to achieve immunomodulatory outcomes.

**Figure 4:**
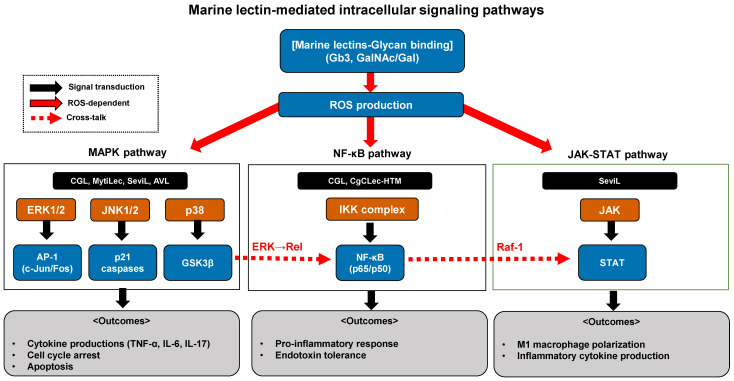
Marine lectin-mediated intracellular signaling pathways and cross-talk mechanisms. Marine lectin–glycan binding (Gb3, GalNAc/Gal) triggers ROS production and activates three major signaling cascades. Pathway characterization derives from both exogenous lectin application to mammalian cells (CGL, SeviL, MytiLec, AVL) and endogenous signaling in bivalve hemocytes (CgCLec-TM1, CgCLec-HTM). The MAPK pathway encompasses three branches (ERK1/2, JNK1/2, p38) that activate distinct transcription factors: AP-1 (c-Jun/Fos) for cytokine transcription, p21 and caspases for cell cycle arrest and apoptosis, and GSK3β for IL-17 expression regulation. The NF-κB pathway proceeds through IKK complex activation and subsequent nuclear translocation of NF-κB (p65/p50), exhibiting context-dependent regulation that results in either pro-inflammatory responses or endotoxin tolerance. The JAK-STAT pathway drives macrophage M1 polarization and inflammatory cytokine production. Pathway cross-talk occurs through ERK-Rel interactions linking MAPK to NF-κB signaling and Raf-1-mediated coordination between NF-κB and JAK-STAT pathways. Marine lectins demonstrating activation of each pathway are indicated in boxes above pathway components. Red arrows indicate ROS-dependent pathway activation; black arrows indicate signal transduction; dashed red lines indicate pathway cross-talk. Abbreviations: AP-1, activator protein-1; AVL, *Aphrocallistes vastus* lectin; CgCLec-HTM, *Crassostrea gigas* C-type lectin with hemITAM motif; CGL, *Crenomytilus grayanus* lectin; ERK, extracellular signal-regulated kinase; GSK3β, glycogen synthase kinase-3β; IKK, inhibitor of κB kinase; IL, interleukin; JAK, Janus kinase; JNK, c-Jun N-terminal kinase; MAPK, mitogen-activated protein kinase; NF-κB, nuclear factor-kappa B; Rel, NF-κB family transcription factor; ROS, reactive oxygen species; SeviL, *Mytilisepta virgata* lectin; STAT, signal transducer and activator of transcription; TNF-α, tumor necrosis factor-alpha.

The aforementioned marine lectin-mediated immunomodulation involves coordinated activation of multiple intracellular signaling pathways with extensive cross-talk mechanisms (Figure 4). The integration of MAPK, NF-κB, and JAK-STAT cascades enables these marine lectins to generate diverse and context-dependent immune responses through ROS-dependent activation and pathway cross-talk.

## 6. Functional Immunological Outcomes

### 6.1. Antibacterial, Antiviral, and Antifungal Activities

Various marine lectins exhibit direct antimicrobial activities that complement their immunomodulatory functions, encompassing both antibacterial and broad-spectrum antiviral effects through membrane perturbation, metabolic pathway inhibition, and viral entry blockade [5]. Antimicrobial peptides CGS19 and CGS20, designed based on fragments derived from sea cucumber hemolytic lectin, exhibited potent activity against clinically isolated methicillin-resistant *Staphylococcus aureus* (MRSA) with minimum inhibitory concentrations of 3–6 μM, achieving 5.9 and 5.8 log reductions in bacterial counts in a mouse subeschar infection model [89]. Mechanistic investigations revealed dual modes of action wherein these peptides disrupted bacterial membrane integrity while simultaneously binding to formate-tetrahydrofolate ligase, a key enzyme in the folate biosynthesis pathway. This dual targeting inhibits folate metabolism essential for bacterial nucleotide synthesis, representing a potentially advantageous approach to circumvent antimicrobial resistance [89].

Some marine lectins demonstrate remarkable broad-spectrum antiviral activity through their capacity to bind oligosaccharide structures on viral envelope glycoproteins and thereby prevent viral entry into host cells [9]. The mannose-binding lectin GRFT, isolated from the red alga *Griffithsia* sp., exhibits particularly potent antiviral activity across diverse viral families, demonstrating efficacy against HIV-1 at picomolar concentrations and showing activity against Zaire Ebola virus with a half-maximal inhibitory concentration (IC50) value of approximately 42 nM [21,40]. Cyanovirin-N from the cyanobacterium *Nostoc ellipsosporum* demonstrated potent activities against HIV-1 and influenza A and B viruses with half-maximal effective concentration (EC50) values of 4–40 nM, and against Ebola virus with EC50 values of 40–60 nM [43,44]. Scytovirin from *Scytonema varium* exhibited EC50 values of 50 nM against Ebola virus with 90% survival in mouse challenge studies. Both lectins bind to mannose-rich oligosaccharides on viral envelope glycoproteins [46]. GRFT functions as an obligate domain-swapped dimer presenting six carbohydrate-binding sites that enable simultaneous binding to multiple high-mannose glycans on viral surfaces. Engineered monomeric GRFT variants exhibit approximately 1000-fold reduced antiviral activity compared to dimeric GRFT despite retaining comparable affinity for isolated oligosaccharides, indicating that cross-linking of viral envelope proteins through multivalent carbohydrate engagement rather than simple glycan occupancy drives potent viral inhibition [90]. Cyanovirin-N similarly requires bivalent binding for antiviral potency. Mutagenesis studies revealed that abolishing carbohydrate binding at domain B completely eliminated antiviral activity, whereas alterations in domain A specificity minimally affected viral inhibition. These findings demonstrate that coordinated engagement of multiple glycan sites on viral glycoproteins constitutes the mechanistic basis for lectin-mediated viral entry blockade [91].

Particular marine lectins exhibit unique capacity for irreversible viral inactivation through mechanisms that extend beyond competitive entry inhibition. Treatment of severe acute respiratory syndrome coronavirus 2 (SARS-CoV-2) pseudovirus with cyanovirin-N or GRFT followed by extensive washing failed to restore viral infectivity. This irreversible inactivation requires multivalent lectin interaction with two distinct glycan clusters on the S1 subunit—one associated with the receptor-binding domain and another with the S1/S2 cleavage site [41]. GRFT retained activity against multiple SARS-CoV-2 variants including Omicron, indicating that lectin-mediated targeting of conserved glycosylation patterns may provide resistance to viral escape mutations that commonly compromise antibody-based therapeutics [41]. GRFT administered to mice provided effective protection against lethal influenza A virus challenge through binding to hemagglutinin glycans, and a bivalent entry inhibitor GL25E comprising GRFT fused to the pan-coronavirus inhibitor peptide EK1 showed enhanced efficacy against SARS-CoV-2 Omicron variants and effectively inhibited in vitro co-infection of influenza A virus and SARS-CoV-2, with favorable safety and stability profiles [42]. In vivo studies demonstrated that cyanovirin-N administered intranasally to mice infected with influenza A virus provided dose-dependent protection with up to 100% survival and 1000-fold reduction in lung virus titers when treatment was initiated early post-infection [45]. GRFT demonstrated protective efficacy in a mouse model of Ebola virus disease with delayed mortality compared to control animals following challenge with mouse-adapted Ebola virus, with pull-down experiments confirming that GRFT binds N-glycans on Ebola virus glycoproteins to block infection, establishing these marine lectins as promising candidates for broad-spectrum antiviral therapeutics targeting conserved glycosylation patterns across diverse viral pathogens [40].

Beyond antiviral activities, several marine lectins demonstrate antifungal properties. Q-Griffithsin (Q-GRFT), a variant of the red algal lectin griffithsin produced in *Nicotiana benthamiana*, exhibited significant preventive and therapeutic activity against *Candida albicans* in murine models of vaginal candidiasis [47]. Experimental analyses indicated that Q-GRFT binds specifically to α-mannan in the fungal cell wall, with this interaction associated with cell wall perturbation and elevated intracellular ROS levels. However, the causal sequence linking mannan engagement to ROS accumulation and fungicidal effects remains incompletely defined, representing an important mechanistic gap relative to the more thoroughly characterized antiviral activities of the parent GRFT lectin [92]. The sea hare egg lectin AKL-40 from *Aplysia kurodai*, when applied exogenously, showed strong antifungal activity against *Talaromyces verruculosus*, in addition to its antibacterial effects against *Staphylococcus aureus*, *Shigella sonnei*, and *Bacillus cereus* [49]. Similarly, mytilectins from the Mytilidae family, including MCL from *Mytilus californianus* and MTL from *Mytilus trossulus*, displayed growth suppressive activities against various fungi [25,26]. The lectin CGL from *Crenomytilus grayanus* inhibited growth of fungi commonly associated with mussels, suggesting a physiological role in host defense against fungal infections [93]. Comparative analysis of antimicrobial mechanisms across the marine lectin structural families presented in Section 2 indicates that specific structural features—particularly multivalency and carbohydrate recognition domain (CRD) architecture—are associated with distinct antimicrobial outcomes through divergent molecular mechanisms (Table 3).

The antiviral lectins GRFT and cyanovirin-N exemplify how multivalent architecture enables potent pathogen inhibition through glycan cross-linking. GRFT functions as an obligate domain-swapped dimer presenting six carbohydrate-binding sites that simultaneously engage multiple high-mannose glycans on viral envelope glycoproteins [90]. This multivalent binding produces potent viral inactivation through envelope protein cross-linking rather than simple competitive inhibition—a mechanism supported by the observation that engineered monomeric GRFT variants exhibit approximately 1000-fold reduced antiviral activity despite retaining comparable affinity for isolated oligosaccharides [90]. Similarly, cyanovirin-N requires bivalent binding for antiviral potency, with mutagenesis studies demonstrating that abolishing carbohydrate binding at domain B completely eliminates antiviral activity [91]. The conserved requirement for multivalency across these structurally distinct lectins establishes glycan cross-linking as a fundamental mechanism for lectin-mediated viral neutralization.

Q-GRFT, which retains the GRFT-based domain-swapped dimeric scaffold with a single amino acid substitution (M78Q), targets α-mannan in fungal cell walls yet produces functionally distinct outcomes compared to its antiviral activity: cell wall perturbation accompanied by elevated intracellular ROS levels rather than the cross-linking and irreversible inactivation observed against viral targets [47,92]. This apparent mechanistic divergence—conserved lectin scaffold engaging different biological contexts—suggests that the physical properties of glycan targets (fluid viral envelope versus rigid fungal cell wall architecture) may influence the downstream consequences of lectin engagement. However, the precise molecular events connecting mannan binding to fungal cell death remain to be fully elucidated, and direct mechanistic comparisons with antiviral activity should be interpreted cautiously pending further characterization.

In contrast, the antibacterial peptides CGS19 and CGS20, designed based on fragments derived from sea cucumber hemolytic lectin sequences [89], do not rely on CRD-mediated glycan recognition and instead employ glycan-independent mechanisms. These peptides achieve bactericidal activity through simultaneous membrane disruption and inhibition of formate-tetrahydrofolate ligase, a key enzyme in folate biosynthesis essential for bacterial nucleotide synthesis [89]. This dual-targeting strategy—combining physical membrane perturbation with metabolic pathway inhibition—represents a mechanistically distinct antimicrobial paradigm from glycan-dependent lectin strategies.

Collectively, these comparisons indicate that marine-derived antimicrobials span a mechanistic spectrum: from glycan-dependent strategies employing multivalent cross-linking (antiviral lectins) or cell wall perturbation with oxidative stress association (antifungal lectins), to glycan-independent approaches combining membrane disruption with enzyme inhibition (lectin-derived antibacterial peptides). The structural classification presented in Section 2 and Table 1 thus provides a framework for understanding how lectin architecture relates to antimicrobial mechanisms, with multivalent CRD-containing lectins favoring glycan-mediated pathogen neutralization and lectin-derived peptides employing alternative, glycan-independent mechanisms. It should be noted that mechanistic resolution varies considerably across these categories: antiviral mechanisms involving envelope cross-linking are well characterized, whereas antifungal mechanisms linking glycan binding to cell death remain at an earlier stage of investigation.

### 6.2. Therapeutic Applications in Inflammatory Diseases

Beyond their antimicrobial functions, marine algal lectins have demonstrated functional anti-inflammatory and analgesic activities in classical in vivo disease models through peripheral mechanisms involving modulation of inflammatory mediators and nociceptive pathways [50]. Lectins isolated from the red marine alga *Solieria filiformis* exhibited significant antinociceptive activity in Swiss mice, reducing acetic acid-induced abdominal writhings by 52% at 72.9 mg/kg intravenous administration and diminishing paw licking time in the second phase of the formalin test by 87%. No significant activity was observed in the hot-plate test, indicating that the antinociceptive action occurs predominantly via peripheral rather than central mechanisms [48]. Similarly, lectin from the red alga *Pterocladiella capillacea* reduced acetic acid-induced writhings by 30%, 39%, and 52% at doses of 0.9, 8.1, and 72.9 mg/kg respectively, and inhibited both the first and second phases of the formalin test by 58% and 87% respectively at 72.9 mg/kg. These antinociceptive effects were abolished when the lectin was pre-incubated with mucin, confirming the carbohydrate-binding specificity underlying the therapeutic action [50].

Marine algal lectins demonstrated potent anti-inflammatory activity in carrageenan-induced inflammation models through reduction in neutrophil migration and edema formation. Lectin from *Pterocladiella capillacea* administered at 8.1 mg/kg intravenously significantly reduced carrageenan-induced neutrophil migration by 84% in male Wistar rats and inhibited paw edema at all time intervals examined. Lectin from the green marine alga CcL reduced carrageenan-induced neutrophil migration by 65.9% at 9 mg/kg and inhibited paw edema at all evaluated time points. These anti-inflammatory effects were abolished when the lectins were pre-incubated with mucin, demonstrating that carbohydrate recognition mediates the therapeutic efficacy [94].

While other marine algae-derived compounds including sulfated polysaccharides, alkaloids, and crude extracts also demonstrate antinociceptive activities, these effects occur through non-glycan-binding mechanisms [95,96,97,98]. In contrast, marine algal lectins are mechanistically distinguished by their glycan-binding specificity.

The antinociceptive mechanisms of marine algal lectins involve modulation of peripheral inflammatory mediators and opioid receptor pathways. The antinociceptive effects of *Solieria filiformis* lectin in both formalin and hot-plate tests were prevented at least partially by pretreatment with the opioid receptor antagonist naloxone at 2 mg/kg subcutaneously [48]. The peripheral selectivity of these marine algal lectins was confirmed through differential responses in thermal versus chemical nociception models, as lectins demonstrated significant activity in acetic acid writhing and the second phase of formalin testing but showed no significant effects in the hot-plate test when compared to morphine, suggesting that the antinociceptive action occurs predominantly through peripheral mechanisms. This conclusion is supported by the observation that lectin effects were abolished upon pre-incubation with mucin, confirming the involvement of carbohydrate-binding domains in mediating the therapeutic effects [50].

### 6.3. Cancer-Associated Immune Effects

Beyond their anti-inflammatory activities, specific marine lectins have demonstrated significant immunotherapeutic potential in cancer contexts through recognition of tumor-associated glycans and modulation of antitumor immune responses. The lectin from the sea mollusk CGL, when applied exogenously, recognizes globotriaosylceramide (Gb3), which is abundantly present on cancer cell surfaces. Binding to Gb3 on the surface of breast cancer cells leads to cell death through a multivalent recognition mechanism enabled by the dimeric quaternary structure that provides six total ligand-binding sites within each CGL dimer, conferring enhanced avidity for Gb3-expressing tumor cells [53]. Similarly, MytiLec isolated from the Mediterranean mussel *Mytilus galloprovincialis*, when applied exogenously, binds strongly to Gb3-containing glycosphingolipid-enriched microdomains on BL cell surfaces, triggering programmed cell death through multiple pathways. These include activation of both classical MAPK/ERK and stress-activated p38 kinase and JNK MAPK pathways, induction of TNF-α expression as a ligand of death receptor-dependent apoptosis, and activation of mitochondria-controlling caspase-9 and caspase-3. These findings demonstrate that tumor glycan recognition by marine lectins initiates coordinated apoptotic and immunogenic signaling cascades [24].

Some marine lectins modulate innate immune cell function to enhance antitumor immunity. The lectin SeviL demonstrated the capacity to induce macrophage polarization toward the M1 functional phenotype when administered to RAW264.7 macrophage cells, positively influencing the expression of several inflammatory molecules with trends markedly different from those observed when mussel lectins were added to cancer cells. These findings indicate that such marine lectins can differentially regulate immune effector cells versus tumor cells through glycan-dependent recognition mechanisms [23]. The immunomodulatory properties of particular marine lectins have been further explored through viral vector delivery systems. Oncolytic vaccinia virus harboring AVL increased the levels of inflammatory cytokines including IL-6, IL-8, and TNF-α through activating the AP-1 signaling pathway in HCC cells, and also upregulated the expression of type I interferons (IFNs) while enhancing virus replication by inhibiting IFN-stimulated response element-mediated viral defense response. These findings suggest that marine lectins delivered via oncolytic vectors can convert immunologically cold tumors into inflamed tumors that exhibit enhanced responsiveness to immunotherapy [10,88].

The tumor glycan recognition capacity of some marine lectins has been exploited for development of lectin-based chimeric antigen receptors. T cells expressing chimeric antigen receptors (CARs) incorporating Gb3-binding lectins including Shiga toxin B-subunit (StxB), LecA from *Pseudomonas aeruginosa*, and the engineered lectin Mitsuba derived from *Mytilus galloprovincialis* as antigen-binding domains demonstrated target-specific cytotoxicity against BL-derived cell lines as well as solid tumor cells from colorectal and triple negative breast cancer. These findings establish that lectin-based CARs represent a viable approach to target tumor-associated carbohydrate antigens expressed in both hematological malignancies and solid tumors [99]. These findings collectively establish the described marine lectins as multifaceted immunotherapeutic agents that engage cancer immunity through tumor glycan recognition leading to immunogenic cell death, direct modulation of innate immune effector cells toward proinflammatory phenotypes, and enhancement of viral oncolytic therapy through coordinated upregulation of inflammatory cytokines and type I IFNs. These properties position marine lectins as promising candidates for development of glycan-targeted immunotherapies that complement existing checkpoint inhibitor and cellular therapy approaches [23,24,53,99,100].

**Figure 5:**
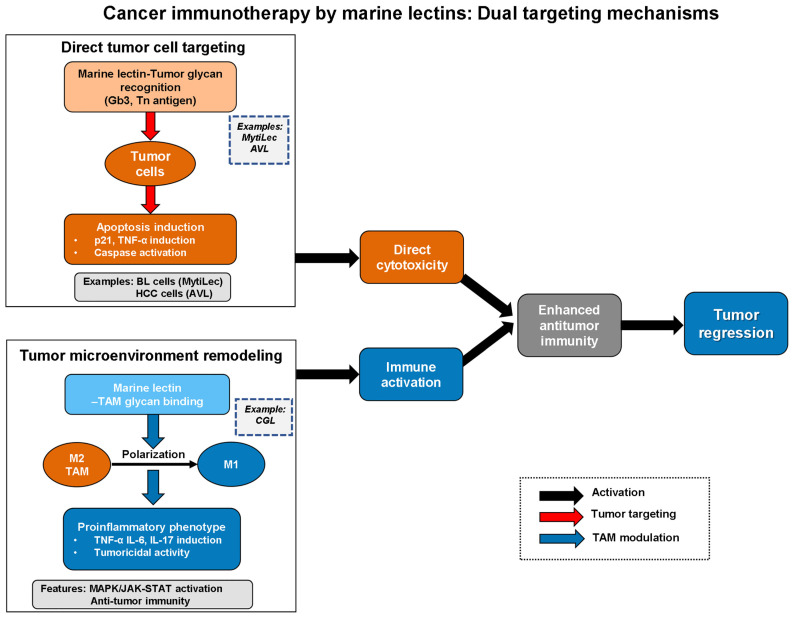
Cancer immunotherapy by marine lectins: Dual targeting mechanisms. Marine lectins exert antitumor effects through two complementary glycan-binding mechanisms: tumor glycan recognition (Gb3, Tn antigen) for direct cytotoxicity and TAM glycan binding for immune activation. Direct tumor cell targeting (**top**): MytiLec and AVL recognize tumor-associated carbohydrate antigens including Gb3 and Tn antigen, inducing apoptosis through upregulation of p21 and TNF-α, and activation of caspase-mediated cell death. MytiLec demonstrates selective cytotoxicity against BL cells via Gb3 recognition, while AVL induces inflammatory responses in HCC cells. Tumor microenvironment remodeling (**bottom**): CGL repolarizes TAMs from immunosuppressive M2 phenotype to pro-inflammatory M1 phenotype, activating MAPK and JAK-STAT signaling pathways and enhancing production of TNF-α, IL-6, and IL-17 with tumoricidal activity. The integration of direct cytotoxicity and immune activation produces enhanced antitumor immunity leading to tumor regression. Arrows indicate: black = activation/induction; red = tumor targeting; orange = TAM modulation. Abbreviations: AVL, *Aphrocallistes vastus* lectin; BL, Burkitt’s lymphoma; CGL, *Crenomytilus grayanus* lectin; Gb3, globotriaosylceramide; HCC, hepatocellular carcinoma; IL, interleukin; JAK-STAT, Janus kinase-signal transducer and activator of transcription; MAPK, mitogen-activated protein kinase; MytiLec, *Mytilus galloprovincialis* lectin; TAM, tumor-associated macrophage; TNF-α, tumor necrosis factor-alpha.

The aforementioned marine lectins target tumors through complementary dual mechanisms (Figure 5): direct tumor cell cytotoxicity via recognition of tumor-associated glycans such as Gb3 (MytiLec, AVL) and tumor microenvironment remodeling through TAM repolarization from M2 to M1 phenotype (CGL). This integrated approach enables synergistic antitumor responses combining direct cytotoxic effects with enhanced immune activation.

## 7. Knowledge Gaps and Future Directions

### 7.1. Adaptive Immunity

While marine lectins demonstrate potent innate immune activation through macrophage stimulation and inflammatory mediator production as described in preceding sections, their effects on adaptive immune responses remain understudied. The algal lectin GRFT provides a notable example of this gap, as studies evaluating its safety profile specifically demonstrated that GRFT treatment does not significantly upregulate markers of T-cell activation including Interleukin-2 receptor alpha chain (CD25), early activation antigen (CD69), and human leukocyte antigen-DR isotype (HLA-DR) on CD4+ and CD8+ T cells, contrasting sharply with other antiviral lectins such as concanavalin A and cyanovirin-N that induce T-cell activation [101]. Similarly, the engineered lectin Q-GRFT showed immunomodulatory effects limited to innate immune cells, with studies in murine vaginal candidiasis models examining only neutrophil and monocyte populations without assessment of lymphocyte responses [47].

Structural analyses have suggested potential interactions between marine lectins and adaptive immune components, yet functional validation remains absent. The sialic acid-binding lectin from *Haliotis discus discus* belongs to the same functional family as Siglecs, which are expressed on B cells and regulate antibody production, yet whether this marine lectin modulates B cell function remains unexplored [39]. The C-type lectin codakine from *Codakia orbicularis* exhibits structural features similar to molecules involved in major histocompatibility complex interactions, yet investigations of its ability to modulate antigen presentation or T cell activation have not been conducted [6].

Marine lectins have been extensively characterized for their direct antimicrobial and cytotoxic activities, with comprehensive reviews documenting their antibacterial, antiviral, and anticancer properties without mention of adaptive immune modulation [5,9]. Even studies specifically focused on immunomodulatory activities of algal lectins have concentrated exclusively on their effects on HIV entry and transmission without examining their impact on T helper cell polarization or cytotoxic T lymphocyte (CTL) responses [102]. This absence of adaptive immunity investigation represents a critical knowledge gap given that effective cancer immunotherapy increasingly relies on coordinating both innate and adaptive immune responses, suggesting that future research should prioritize examination of marine lectin effects on DC maturation, antigen cross-presentation, T cell differentiation, B cell activation, and immunological memory formation.

### 7.2. Detailed Cellular Mechanisms

The molecular mechanisms underlying most marine lectin interactions with professional antigen-presenting cells remain virtually unexplored despite the critical importance of these cells in bridging innate and adaptive immunity. While the C-type lectin codakine from *Codakia orbicularis* exhibits structural homologies with human endothelial selectin (E-selectin) and murine NK cell lectins that interact with major histocompatibility complex (MHC) molecules for opsonization and phagocytosis, no functional studies have examined whether this marine lectin actually modulates antigen presentation or DC maturation [6]. This structural similarity without functional validation exemplifies the current knowledge gap in understanding how marine lectins might influence the cellular machinery of immune activation.

NK cells, which serve as critical mediators between innate and adaptive immunity through their cytotoxic functions and cytokine production, have not been investigated as targets of marine lectin immunomodulation. Despite extensive characterization of marine lectin cytotoxicity against various cancer cell lines including HCC, cervical cancer, and melanoma cells, none of the studies examined effects on NK cell activation, proliferation, or cytotoxic function [8]. Similarly, regulatory T cells (Tregs), which play essential roles in maintaining immune homeostasis and preventing autoimmunity [103,104], have not been studied in the context of marine lectin exposure, leaving a critical gap in understanding whether these molecules could modulate immune tolerance or suppression.

The absence of research on marine lectin effects on antigen-presenting cell (APC) function is particularly notable, as comprehensive reviews of their biological activities focus exclusively on direct antimicrobial, antiviral, and anticancer properties without addressing immunological mechanisms involving DCs, NK cells, or Tregs [5]. This gap suggests that future investigations should prioritize examination of individual marine lectin effects on DC phenotype markers, antigen processing and presentation pathways, NK cell receptor expression, cytotoxic granule release, and Treg differentiation and suppressive function to fully understand their immunomodulatory potential.

### 7.3. Clinical Translation Needs

Despite the promising antimicrobial activities of certain marine lectins, comprehensive preclinical and clinical safety evaluations remain paramount for product development. Safety concerns with some lectins, including mitogenic activity after prolonged exposures and challenges in mass production, continue to impact clinical progression [9]. Among marine lectins evaluated for clinical applications, the algal lectin GRFT represents the most extensively characterized candidate, with preclinical safety studies demonstrating a no-observed-adverse-effect level at 0.3% concentration in gel formulations applied to rabbits, with minimal vaginal irritation and little or no systemic detection following topical administration [105]. Formulation development for GRFT has progressed to include both gel-based vehicles and fast-dissolving inserts designed for vaginal delivery, with the latter demonstrating protective efficacy against herpes simplex virus (HSV)-2 infection in murine models when administered four hours prior to viral challenge [105].

The most clinically advanced applications of viral vectors for cancer therapy include JX-594, a thymidine kinase-deleted oncolytic vaccinia virus expressing granulocyte-macrophage colony-stimulating factor (GM-CSF) that has advanced to phase III clinical trials for hepatocellular carcinoma (HCC) treatment, and the related vaccinia virus GL-ONC1, which has similarly progressed to clinical trials and demonstrated efficacy in head and neck squamous-cell carcinoma (HNSCC). In contrast, oncolytic vaccinia viruses armed with marine lectins including AVL remain in preclinical development stage [73]. Recombinant mistletoe lectin (ML), derived from terrestrial rather than marine sources, has achieved phase I clinical testing with positive tumor stabilization results, providing proof-of-concept that lectin-based therapeutics can advance to human trials [5].

The fundamental barriers to clinical translation of many marine lectins include expensive production requiring complex purification procedures that complicate scaling-up to commercial manufacturing levels, poor oral bioavailability necessitating alternative delivery routes, potential for hemagglutination of human red blood cells at therapeutic concentrations, mitogenic activity that may stimulate unwanted cellular proliferation, direct cellular toxicity to normal cells in addition to target cells, and induction of differentiation and activation markers on immune cells that could promote immunogenicity, with the cyanobacterial lectin cyanovirin exemplifying several of these compromising properties that have impeded clinical development despite potent antiviral activity [106].

### 7.4. Cancer Immunotherapy Potential

The TME presents a major barrier to effective cancer immunotherapy, with TAMs typically exhibiting M2-like phenotypes that support tumor growth, and reprogramming these macrophages toward M1 phenotypes has emerged as a therapeutic strategy to restore antitumor immunity [107,108]. Hot tumors exhibit strong responses to immunotherapy while cold tumors demonstrate striking features of T cell absence or exclusion, prompting investigation of strategies to convert cold tumors into hot tumors to improve therapeutic efficacy [109,110]. Certain marine lectins’ demonstrated capacity to induce macrophage polarization toward pro-inflammatory M1 functional phenotypes [23] suggests potential utility in reprogramming the immunosuppressive tumor microenvironment, yet systematic investigation of this application remains absent.

The most direct evidence for marine lectin-based cancer immunotherapy derives from oncolytic vaccinia virus harboring AVL, which activates tumor immunity by upregulating type I IFN expression while enhancing virus replication, and increases inflammatory cytokine levels including IL-6, IL-8, and TNF-α in HCC cells [88]. An alternative approach employs lectin-based chimeric antigen receptors, with Gb3-binding lectins demonstrating target-specific cytotoxicity against BL and solid tumors from colorectal and triple-negative breast cancer (TNBC) [99].

Critical knowledge gaps impede clinical translation. No studies have evaluated these marine lectins in combination with checkpoint inhibitors, despite rationale for combining tumor-associated macrophage-targeting therapies with immune checkpoint inhibitors to enhance immune responses [107] and lectins’ demonstrated M1-polarizing effects [23]. Most fundamentally, while viral vector-delivered marine lectins demonstrate TME remodeling capabilities [88], whether purified marine lectins alone can achieve comparable effects has not been investigated. These knowledge gaps are further compounded by taxonomic limitations in the current literature.

### 7.5. Taxonomic Scope and Evidence Landscape

The mechanistic synthesis in Section 3, Section 4 and Section 5 (and the pathway-oriented summary in Table 2) is necessarily dominated by bivalve-derived lectins, particularly members of the Mytilectin family, because the current literature provides signaling-level resolution (e.g., MAPK/NF-κB/JAK-STAT activation, defined transcriptional outputs, and tolerance phenotypes) for only a limited subset of marine lectins. For lectins from other marine taxa, published evidence more commonly establishes glycan-binding specificity and phenotypic outcomes (e.g., pathogen binding, viral entry blockade, antimicrobial effects, or apoptosis in heterologous cell systems) without delineating upstream immune signaling intermediates in native immune contexts (Table 4). Accordingly, conclusions regarding conserved intracellular signaling logic are drawn primarily from lectins with pathway-level evidence, whereas cross-taxa comparisons are explicitly restricted to the evidence-supported levels and should not be interpreted as implying equivalent mechanistic resolution across all marine lectin taxa. Addressing this imbalance—by resolving signaling intermediates for non-bivalve lectins in relevant immune systems—remains a priority for future marine lectin immunology research. While lectins have been identified across diverse marine phyla including echinoderms, cnidarians, ascidians, and fish, most studies on non-bivalve marine lectins have focused on structural characterization, carbohydrate-binding specificity, or direct antimicrobial activities without detailed investigation of intracellular signaling cascades. Fish lectins such as *Dicentrarchus labrax* fucose-binding lectin (DlFBL) and *Strongylocentrotus purpuratus* rhamnose-binding lectin (SpRBL) have demonstrated MAPK/ERK pathway activation, but these findings derive from exogenous expression in mammalian cancer cells rather than native fish immune responses [38]. Similarly, cnidarian and echinoderm immune studies have identified lectin involvement in pathogen recognition and cytokine-like responses, yet the specific signaling intermediates linking lectin engagement to effector functions remain largely uncharacterized. Future research should prioritize mechanistic studies of lectin-mediated immune signaling across broader taxonomic groups to enable more comprehensive comparative analysis of marine lectin immunomodulation.

## 8. Conclusions

The lectins reviewed herein with signaling-level mechanistic evidence—currently dominated by bivalve-derived lectins— function as pattern recognition molecules that couple glycan recognition to intracellular pathway activation. Mechanistic evidence derives from two experimental contexts: endogenous lectin functions within source organisms (e.g., CgCLec-TM1/HTM in oyster hemocytes recognizing bacterial pathogens) and exogenous application of purified lectins to mammalian cell models (e.g., CGL, SeviL, MytiLec activating macrophage signaling). Notably, some lectins such as CGL induce immunomodulation through CRD-independent mechanisms, indicating that non-glycan-binding domains may also contribute to immune activation. Within this evidence-supported subset, activation of MAPK, NF-κB, and JAK-STAT cascades drives diverse immunomodulatory outcomes including cytokine regulation, immune cell polarization, endotoxin tolerance, and apoptosis induction. For marine lectins from other taxa, comparable signaling resolution is frequently unavailable, and mechanistic generalizations should therefore be interpreted as evidence-limited pending future pathway-level studies in relevant immune contexts.

However, critical knowledge gaps limit understanding of their immunomodulatory mechanisms and impede clinical translation. Specific marine lectin effects on adaptive immune responses, including DC maturation, T cell differentiation, and B cell activation, remain largely uncharacterized. Interactions with NK cells and regulatory T cells have not been systematically investigated. Clinical development faces barriers including complex purification requirements, limited bioavailability, potential off-target effects, and absence of human safety and efficacy data. The capacity of these marine lectins to modulate the TME and enhance cancer immunotherapy in combination with checkpoint inhibitors has not been evaluated despite their demonstrated effects on macrophage polarization.

Future investigations should characterize individual marine lectin effects on adaptive immunity, evaluate combination strategies with established immunotherapies, develop scalable production methods, and assess safety and efficacy in preclinical models. The marine lectins described in this review warrant continued investigation as immunomodulatory agents, with systematic research needed to determine their therapeutic potential and establish appropriate clinical applications.

## Figures and Tables

**Table 2:** Marine lectins with pathway-level mechanistic evidence for immunomodulatory activity. Selected examples of marine lectins for which intracellular signaling intermediates (e.g., MAPK/NF-κB/JAK-STAT modules) have been experimentally characterized, alongside source taxa, glycan specificity, and key immune-related outcomes. The predominance of bivalve-derived lectins reflects the current state of mechanistic research rather than selective exclusion; non-bivalve entries are included where signaling resolution is available. Arrows (→) represent sequential activation in signaling cascades. Abbreviations: AP-1, activator protein-1; AVL, *Aphrocallistes vastus* lectin; CgCLec-HTM, *Crassostrea gigas* C-type lectin with hemITAM motif; CgCLec-TM1, *Crassostrea gigas* C-type lectin transmembrane protein 1; CGL, *Crenomytilus grayanus* lectin; ERK, extracellular signal-regulated kinase; GalNAc/Gal, galactose/N-acetylgalactosamine; Gb3, globotriaosylceramide; GRFT, griffithsin; GSK3β, glycogen synthase kinase-3β; HCC, hepatocellular carcinoma; HIV, human immunodeficiency virus; IL, interleukin; JAK-STAT, Janus kinase-signal transducer and activator of transcription; JNK, c-Jun N-terminal kinase; LPS, lipopolysaccharide; Man-9, mannose-9; MAPK, mitogen-activated protein kinase; MEK, MAPK; MytiLec, *Mytilus galloprovincialis* lectin; NF-κB, nuclear factor-kappa B; PKC, protein kinase C; Rel, NF-κB family transcription factor; ROS, reactive oxygen species; SARS-CoV-2, severe acute respiratory syndrome coronavirus 2; SeviL, *Mytilisepta virgata* lectin; Syk, spleen tyrosine kinase; TNF-α, tumor necrosis factor-alpha.

Lectin	Source	Glycan Specificity	Signaling Pathways	Key Immune Effects
CGL [22]	*Crenomytilus grayanus* (mussel)	GalNAc/Gal	ROS → MAPK (ERK, JNK, p38), PKC-α/б, NF-κB	TNF-α, IL-6 production; Endotoxin tolerance
SeviL [23]	*Mytilisepta virgata* (mussel)	Galactoside	MAPK, JAK-STAT	M1 macrophage polarization
AVL [10,33]	*Aphrocallistes vastus* (sponge)	D-galactose	MAPK/ERK → AP-1	IL-6, IL-8, TNF-α in HCC cells
MytiLec [24]	*Mytilus galloprovincialis* (mussel)	Gb3	MAPK (MEK-ERK, p38, JNK)	Apoptosis via Gb3 binding; TNF-α, p21 induction
CGCLec-TM1 [27]	*Crassostrea gigas* (oyster)	LPS, bacteria	Syk → ERK → GSK3β	IL-17-1, IL-17-5 expression
CGCLec-HTM [28]	*Crassostrea gigas* (oyster)	LPS, bacteria	Syk → ERK → Rel → NF-κB	IL-17, TNF production
GRFT [21,40,41,42]	*Griffithsin* sp. (red alga)	High-mannose (Man-9)	Envelope glycan binding	Antiviral (HIV, Ebola, SARS-CoV-2, influenza)
Cyanovirin-N [43,44,45]	*Nostoc ellipsosporum* (cyanobacterium)	Mannose-rich	Domain B glycan binding	Antiviral (HIV, Ebola, influenza)

**Table 3:** Antimicrobial activities of marine lectins and lectin-derived peptides. Summary of marine lectins and derived peptides characterized for antibacterial, antiviral, and antifungal activities, showing their taxonomic sources, glycan binding specificities, target pathogens, activity values, and in vivo efficacy data. The entries are organized according to three dominant mechanistic categories reported in the cited literature: (1) multivalent glycan cross-linking of viral envelope glycoproteins associated with irreversible inactivation (GRFT, cyanovirin-N, scytovirin); (2) fungal cell wall α-mannan binding associated with cell wall perturbation and ROS elevation (Q-GRFT); and (3) glycan-independent dual targeting through membrane disruption combined with metabolic enzyme inhibition (CGS19/CGS20). These mechanistic categories correspond to the structural features described in Table 1 and Section 2; evidence depth varies across categories as discussed in the text. EC50, half-maximal effective concentration; GRFT, griffithsin; HIV-1, human immunodeficiency virus type 1; IC50, half-maximal inhibitory concentration; MIC, minimum inhibitory concentration; MRSA, methicillin-resistant *Staphylococcus aureus*; SARS-CoV-2, severe acute respiratory syndrome coronavirus 2; AKL-40, *Aplysia kurodai* lectin-40; Q-GRFT, Q-Griffithsin.

Lectin	Source	Glycan Specificity	Target Pathogen	Activity	In Vivo Efficacy	Ref.
CGS19/CGS20	Sea cucumber hemolytic lectin (derived peptides)	Glycan-independent	MRSA	MIC 3–6 μM	5.8–5.9 log reduction (mouse subeschar)	[89]
GRFT	*Griffithsia* sp. (red alga)	High-mannose (Man-9)	HIV-1	Picomolar	-	[21]
			Ebola	IC50~42 nM	Delayed mortality (mouse)	[40]
			SARS-CoV-2	Active (incl. Omicron)	Irreversible inactivation	[41]
			Influenza A	-	Protection (mouse)	[42]
Cyanovirin-N	*Nostoc ellipsosporum* (cyanobacterium)	Mannose-rich	HIV-1	EC50 4–40 nM	-	[43]
			Influenza A/B	EC50 4–40 nM	100% survival, 1000× viral reduction (mouse)	[43,45]
			Ebola	EC50 40–60 nM	-	[44]
Scytovirin	*Scytonema varium* (cyanobacterium)	Mannose-rich	Ebola	EC50 50 nM	90% survival (mouse)	[46]
Q-GRFT	*Nicotiana benthamiana* (recombinant)	High-mannose	*Candida albicans*	Antifungal	Reduced fungal burden (mouse vaginal candidiasis)	[47]
AKL-40	*Aplysia kurodai* (sea hare)	D-galacturonic acid, D-galactose	*Talaromyces verruculosus*, *Staphylococcus aureus*, *Bacillus cereus*	Antifungal, Antibacterial	-	[49]

**Table 4:** Comparative evidence levels for marine lectin immunomodulation across taxa. Summary of mechanistic evidence depth by taxonomic group. Evidence levels are defined as follows: High = systematic characterization of intracellular signaling cascades (upstream receptor engagement → defined intermediates such as MAPK/NF-κB/JAK-STAT → transcriptional outputs/cytokine production) in immune cells; Medium = functional immunological effects (e.g., cytokine modulation, antimicrobial activity, apoptosis induction) demonstrated without resolution of upstream signaling intermediates; Low = structural, glycan-binding, or phenotypic characterization predominant, with signaling pathway analysis not systematically resolved in immune-cell contexts. “Not systematically resolved in immune-cell contexts” indicates that while some data may exist, systematic pathway characterization is lacking. AJL1, *Anguilla japonica* lectin 1; AjCTL-1, *Apostichopus japonicus* C-type lectin-1; AKL-40, *Aplysia kurodai* lectin-40; APL, *Asterina pectinifera* lectin; AP-1, activator protein-1; AVL, *Aphrocallistes vastus* lectin; CcL, *Caulerpa cupressoides* lectin; CgCLec-HTM, *Crassostrea gigas* C-type lectin with hemITAM motif; CgCLec-TM1, *Crassostrea gigas* C-type lectin transmembrane protein 1; CGL, *Crenomytilus grayanus* lectin; Cyanovirin-N, cyanovirin-N (cyanobacterial lectin from *Nostoc ellipsosporum*); DlFBL, *Dicentrarchus labrax* fucose-binding lectin; ERK, extracellular signal-regulated kinase; GRFT, griffithsin; GSK3β, glycogen synthase kinase-3β; HddSBL, *Haliotis discus discus* sialic acid-binding lectin; JAK–STAT, Janus kinase–signal transducer and activator of transcription; MAPK, mitogen-activated protein kinase; MytiLec, *Mytilus galloprovincialis* lectin; NF-κB, nuclear factor kappa B; PcL, *Pterocladiella capillacea* lectin; SeviL, *Mytilisepta virgata* lectin; SfL, *Solieria filiformis* lectin; SpRBL, *Strongylocentrotus purpuratus* rhamnose-binding lectin; Scytovirin, scytovirin (cyanobacterial lectin from *Scytonema varium*); Syk, spleen tyrosine kinase; TTL, *Tachypleus tridentatus* lectin; WCL, white-spotted charr lectin.

Taxonomic Group	Representative Lectins	Signaling Pathways	Evidence Level	Key Gaps	Ref.
Bivalve (Mytilidae)	CGL, MytiLec, SeviL	MAPK, NF-κB, JAK-STAT	High	Adaptive immunity	[22,23,24,111]
Bivalve (Ostreidae)	CgCLec-TM1, CgCLec-HTM	Syk → ERK → GSK3β	High	Mammalian validation	[27,28]
Sponges	AVL, Halilectin-3	MAPK/ERK, AP-1	Medium-High	Native immune cells	[10,33,67,112]
Red algae	GRFT, PcL, SfL	Not systematically resolved in immune-cell contexts	Medium	Signaling mechanisms	[21,48,50]
Green algae	CcL	Not systematically resolved in immune-cell contexts	Medium	Cellular mechanisms	[51]
Cyanobacteria	Cyanovirin-N, Scytovirin	Not systematically resolved in immune-cell contexts	Low-Medium	Immune cell interactions	[43,44,45,46]
Fish	DlFBL, AJL1, WCL	MAPK (cancer cells only)	Low-Medium	Native fish immune signaling	[35,36,37,38]
Echinoderms	AjCTL-1, SpRBL, APL	MAPK (heterologous expression)	Low	Native immune signaling	[34,35,38]
Gastropods	HddSBL, AKL-40	Not systematically resolved in immune-cell contexts	Low	Immunomodulation	[39,49]
Horseshoe crabs	TTL	Not systematically resolved in immune-cell contexts	Low	Signaling pathways	[35]

## Data Availability

Data are contained within the article.

## Abbreviations

The following abbreviations are used in this manuscript:

3′-UTRs	3′-untranslated regions
ABTS	2,2′-Azino-bis(3-ethylbenzothiazoline-6-sulfonic acid)
ADAM17	A Disintegrin And Metalloprotease 17
AiL	*Argopecten irradians* lectin
AjCTL-1	*Apostichopus japonicus* C-type lectin-1
AJL1	*Anguilla japonica* lectin 1
AKL-40	*Aplysia kurodai* lectin-40
Akt	Protein kinase B
AmL	*Amansia multifida* lectin
AMPK	AMP kinase
AP-1	Activator protein-1
APC	Antigen-presenting cell
APL	*Asterina pectinifera* lectin
AVL	*Aphrocallistes vastus* lectin
Bcl-2	B-cell lymphoma 2
BJcuL	*Bothrops jararacussu* venom-derived galactose-binding lectin
BL	Burkitt’s lymphoma
BMDMs	Bone marrow-derived macrophages
BRG1	Brahma-related gene 1
CARs	Chimeric antigen receptors
CcL	*Caulerpa cupressoides* lectin
CD25	Interleukin-2 receptor alpha chain
CD69	Early activation antigen CD69
CfL	*Chlamys farreri* lectin
CgCLec-HTM	*Crassostrea gigas* C-type lectin with hemITAM motif
CgCLec-TM1	*Crassostrea gigas* C-type lectin transmembrane protein 1
CgIL-17-1	*Crassostrea gigas* IL-17 isoforms CgIL-17-1
CgIL-17-5	*Crassostrea gigas* IL-17 isoform 5
CGL	*Crenomytilus grayanus* lectin
COX	Cyclooxygenase
CpG	Cytosine-phosphate-guanine dinucleotide
CTLA-4	Cytotoxic T lymphocyte
DCs	Dendritic cells
DlFBL	Dicentrarchus labrax fucose-binding lectin
DPPH	2,2-Diphenyl-1-picrylhydrazyl
E2F-1	E2F transcription factor 1
EC50	Half-maximal effective concentration
ERK	Extracellular signal-regulated kinase
E-selectin	Endothelial selectin
GalNAc/Gal	Galactose/N-acetylgalactosamine
Gb3	Globotriaosylceramide
GM-CSF	Granulocyte-macrophage colony-stimulating factor
GRFT	Griffithsin
GSK3β	Glycogen synthase kinase-3β
H3K9	Histone H3 lysine 9
Halilectin-3	Lectin from the marine sponge *Haliclona caerulea*
HCC	Hepatocellular carcinoma
HDACs	Histone deacetylases
HddSBL	*Haliotis discus discus* sialic acid-binding lectin
hemITAM	Half immunoreceptor tyrosine-based activation motif
HIV-1	Human immunodeficiency virus type 1
HLA-DR	Human leukocyte antigen—DR isotype
HNSCC	Head and neck squamous-cell carcinoma
HP1α	Heterochromatin protein 1 alpha
HSV	Herpes simplex virus
IC50	Half-maximal inhibitory concentration
ICAM-1	Intercellular adhesion molecule-1
ICB	Immune checkpoint blockade
IFN	Interferon
IKK	IκB kinase
IL	Interleukin
iNOS	Inducible nitric oxide synthase
IRAK	Interleukin-1 receptor-associated kinase
IκB	Inhibitor of κB
ITAM	Immunoreceptor tyrosine-based activation motif
JAK	Janus kinase
JNK	c-Jun N-terminal kinase
LOX	Lipoxygenase
LPS	Lipopolysaccharide
MAPK	Mitogen-activated protein kinase
McIL-17-1	*Mytilus coruscus* IL-17 isoform 1
MCL	*Haliotis discus discus* sialic acid-binding lectin
MDMs	Blood monocyte-derived macrophages
MEK	Mitogen-activated protein kinase
MgC1q	*Mytilus galloprovincialis* C1q domain-containing protein
MHC	Major histocompatibility complex
miR	MicroRNA
MkC1qDC	*Modiolus kurilensis* C1q domain-containing lectin
ML	Mistletoe lectin
MPO	Myeloperoxidase
MRSA	Methicillin-resistant *Staphylococcus aureus*
MST	Mammalian STE20-like protein kinase
MTL	*Mytilus trossulus* lectin
MytiLec	*Mytilus galloprovincialis* lectin
NF-κB	Nuclear factor kappa B
NK	Natural killer
NO	Nitric oxide
OAA	*Oscillatoria agardhii* agglutinin
OAAH	*Oscillatoria agardhii* agglutinin homolog
PAMPs	Pathogen-associated molecular patterns
PBMCs	Peripheral blood mononuclear cells
PcL	*Pterocladiella capillacea* lectin
PD-1	Programmed cell death protein 1
PD-L1	Programmed death-ligand 1
PGN	Peptidoglycan
PI3K	Phosphatidylinositol 3-kinase
PKC	Protein kinase C
PRRs	Pattern recognition receptors
Q-GRFT	Q-Griffithsin
Rel	NF-κB family transcription factor Rel
ROS	Reactive oxygen species
SARS-CoV-2	Severe acute respiratory syndrome coronavirus 2
Scytovirin	Cyanobacterial lectin from *Scytonema varium*
SeviL	Galactoside-binding lectin from *Mytilisepta virgata*
SfL	*Solieria filiformis* lectin
SHIP1	Src homology 2 domain-containing inositol 5′-phosphatase 1
SOCS1	Suppressor of cytokine signaling 1
SpRBL	*Strongylocentrotus purpuratus* rhamnose-binding lectin
STAT	Signal transducer and activator of transcription
StxB	Shiga toxin B-subunit
Syk	Spleen tyrosine kinase
TAMs	Tumor-associated macrophages
TLR	Toll-like receptor
TME	Tumor microenvironment
TNBC	Triple-negative breast cancer
TNF-α	Tumor necrosis factor alpha
Tregs	Regulatory T cells
TTL	*Tachypleus tridentatus* lectin
VCAM-1	Vascular cell adhesion molecule-1
WCL	White-spotted charr lectin
XIAP	X-linked inhibitor of apoptosis protein
XO	Xanthine oxidase
